# Supplementary material for: Cryo-EM structure of the PlexinC1/A39R complex reveals inter-domain interactions critical for ligand-induced activation
Source: Nat Commun. 2020 Apr 23;11:1953. doi: 10.1038/s41467-020-15862-0 (PMC7181871; doi:10.1038/s41467-020-15862-0)
Supplement: Supplementary file 1 — Supplementary Information [file 41467_2020_15862_MOESM1_ESM.pdf]

Supplementary Information

Cryo-EM Structure of the PlexinC1/A39R complex reveals inter-domain interactions critical for ligand-induced activation

Kuo et al

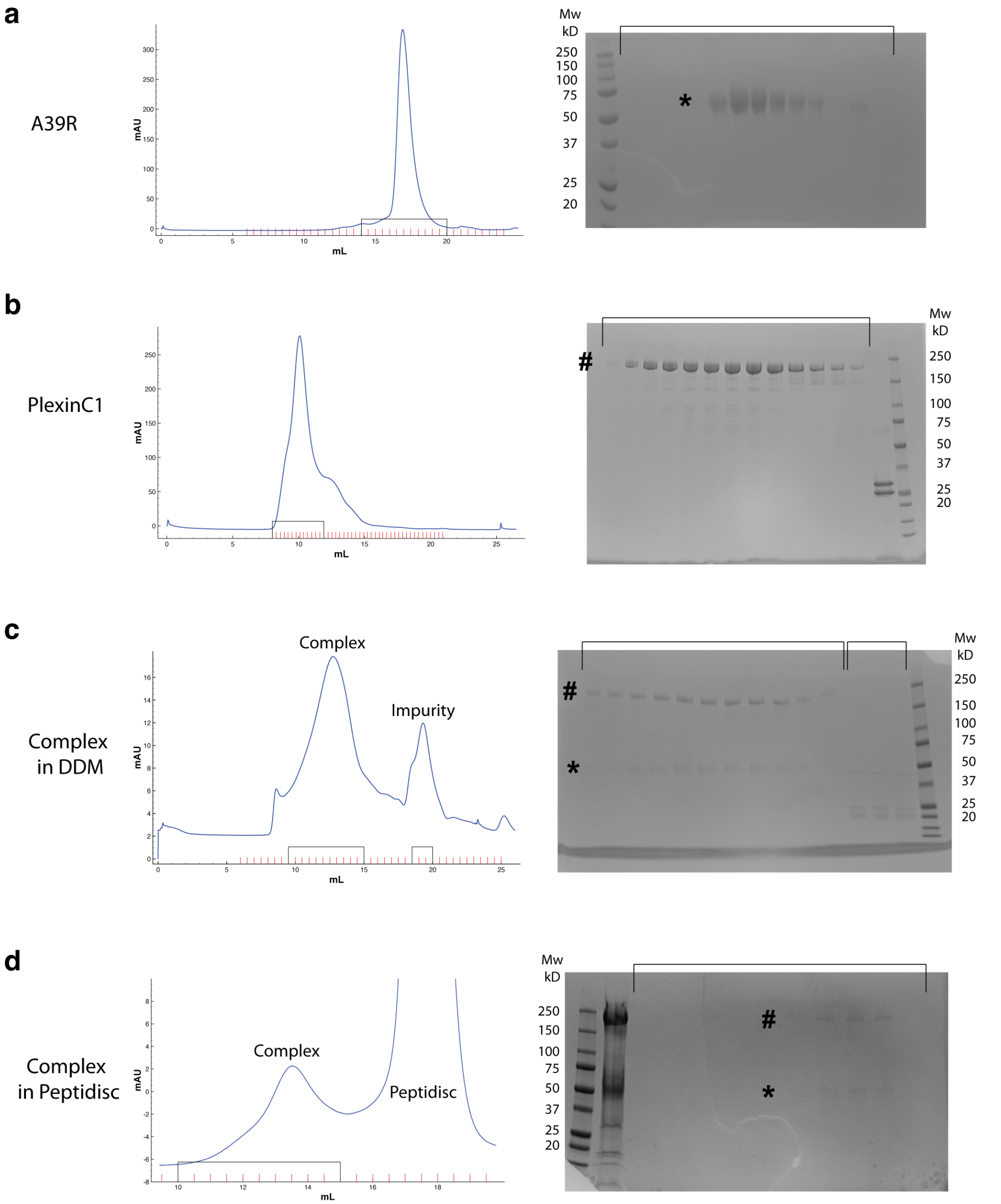

Supplementary Figure 1. Purification of PlexinC1 and A39R and reconstitution of the complex

(a) Gel filtration (Superose 6) and gel analyses of A39R in buffer C.

(b) Gel filtration (Superdex 200) and gel analyses of human PlexnC1 in DDM-containing buffer.

(c) Gel filtration (Superose 6) and gel analyses the PlexinC1/A39R complex in DDM-containing buffer.

(d) Gel filtration (Superose 6) and gel analyses the PlexinC1/A39R complex reconstituted in peptidisc without detergent. Fractions eluted at 12.5-14.5 ml were pooled as cryo-EM sample.

and * indicate the PlexinC1 and A39R bands, respectively.

For all the panels, one representative result from at least three independent experiments is shown.

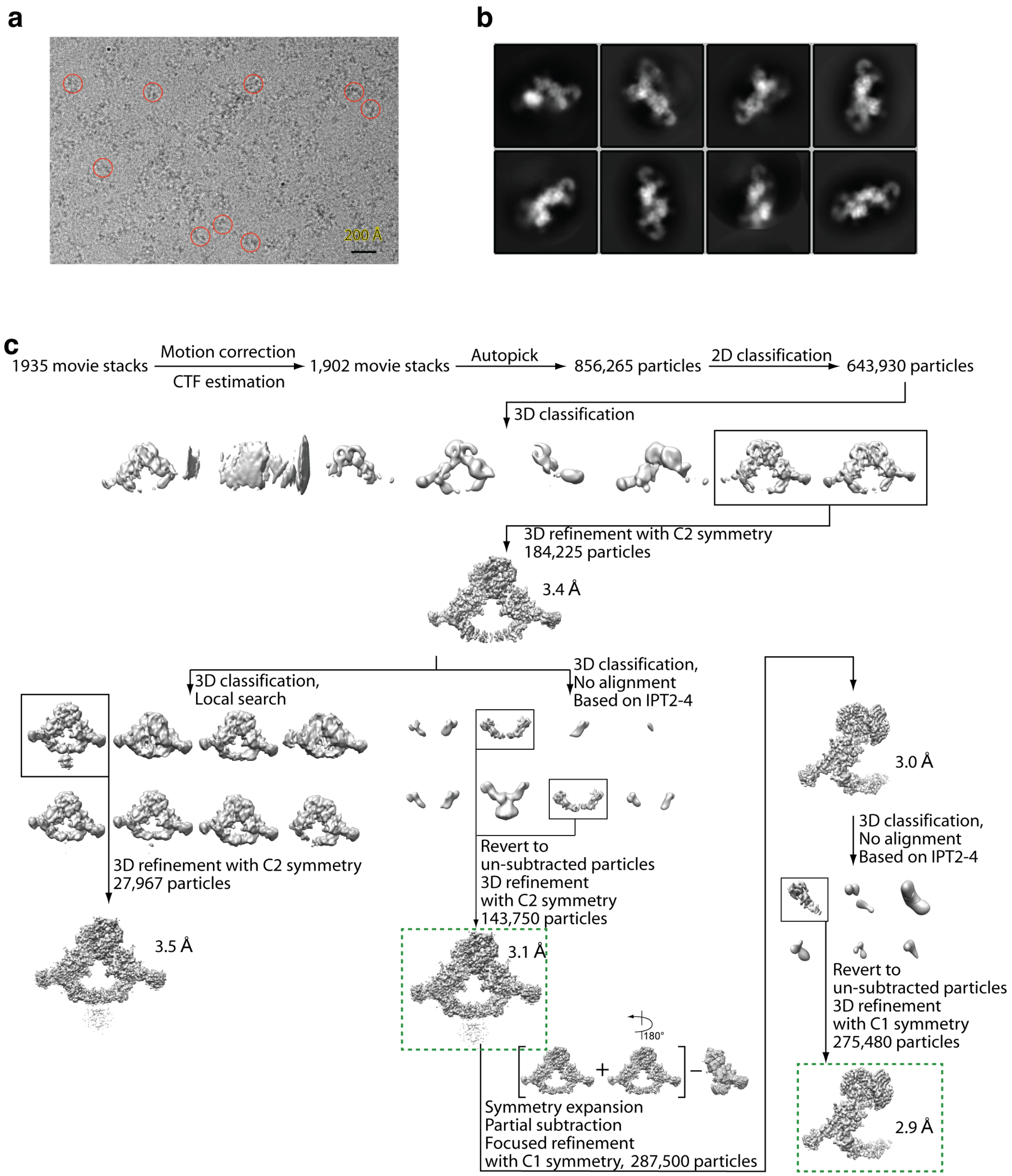

Supplementary Figure 2. Image processing procedure of the PlexinC1/A39R complex
(a) One representative motion-corrected micrograph from the 1935 movie stacks. A few particles are highlighted by circles.
(b) All eight 2D class averages of particles used for 3D reconstruction.
(c) Flow-chart of the image processing procedure. The two maps highlighted by the green rectangles are used for calculating local resolutions and model building.

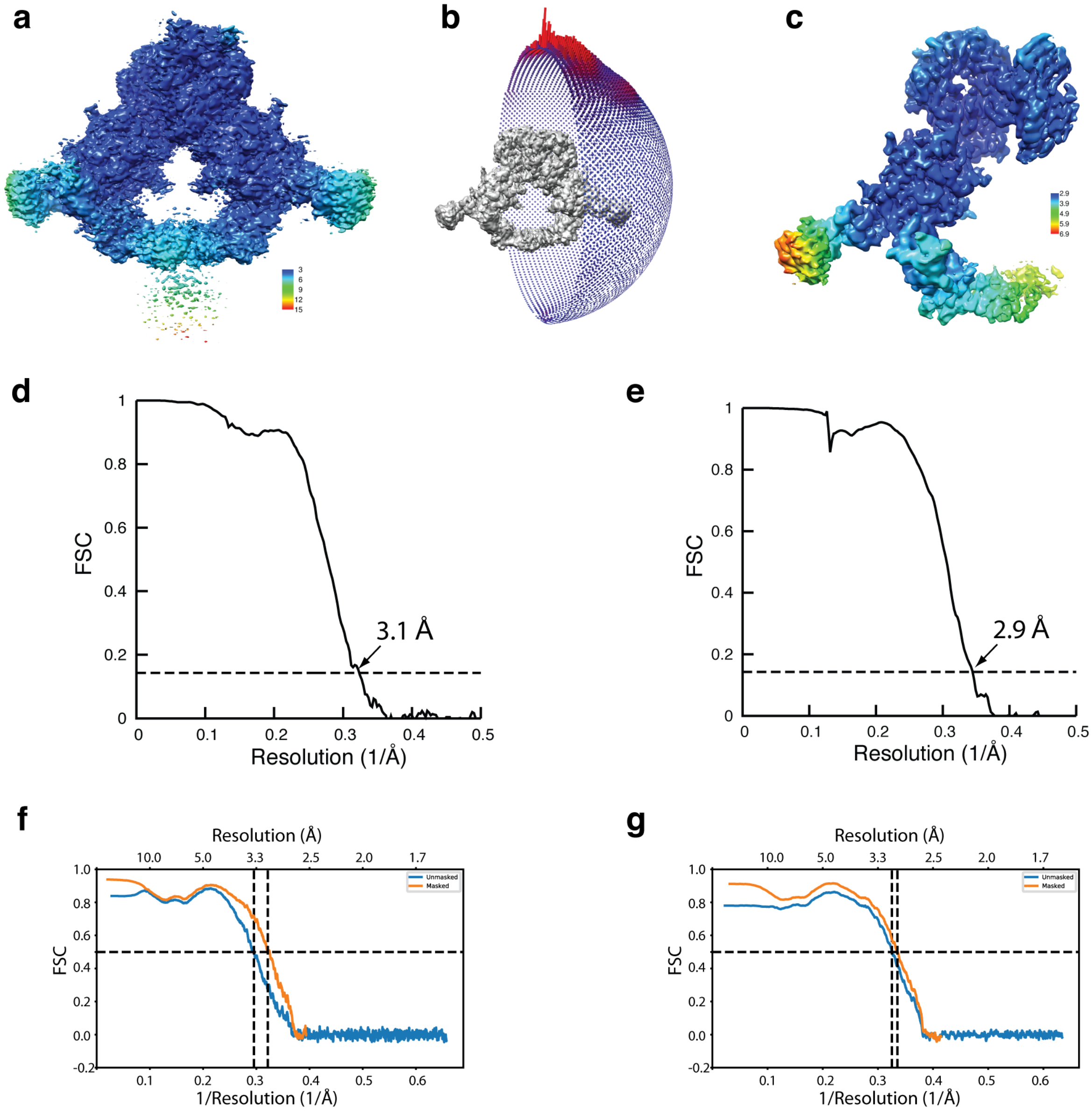

Supplementary Figure 3. Overall and local resolutions of maps.

(a) and (c) Local resolutions of the intact map of the dimeric complex and the focused refined half map, respectively. The scales bars show the resolution ranges in Å.

(b) Euler angle distribution of particles used for the 3D reconstruction of the dimer complex. Height of rods represents the number of particles.

(d) and (e) Gold standard FSC curves of the final reconstructions of the dimeric complex and the focused refined half map, respectively. Dash lines indicate FSC=0.143.

(f) and (g) FSC curves between the maps and models for the intact complex and the focused refined half model, respectively. Dash lines indicate FSC=0.5.

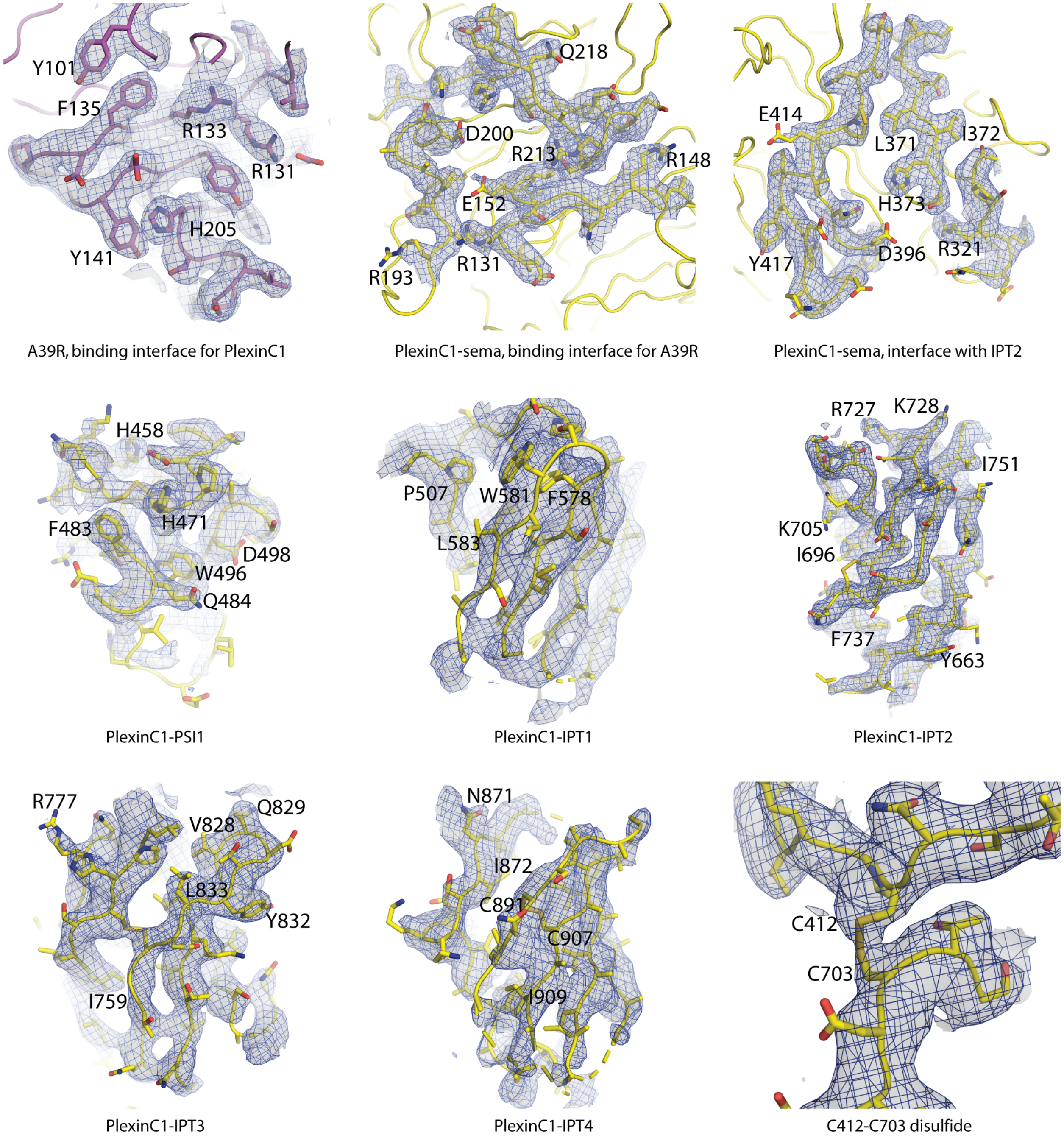

Supplementary Figure 4. Representative density maps of various parts of the structure.

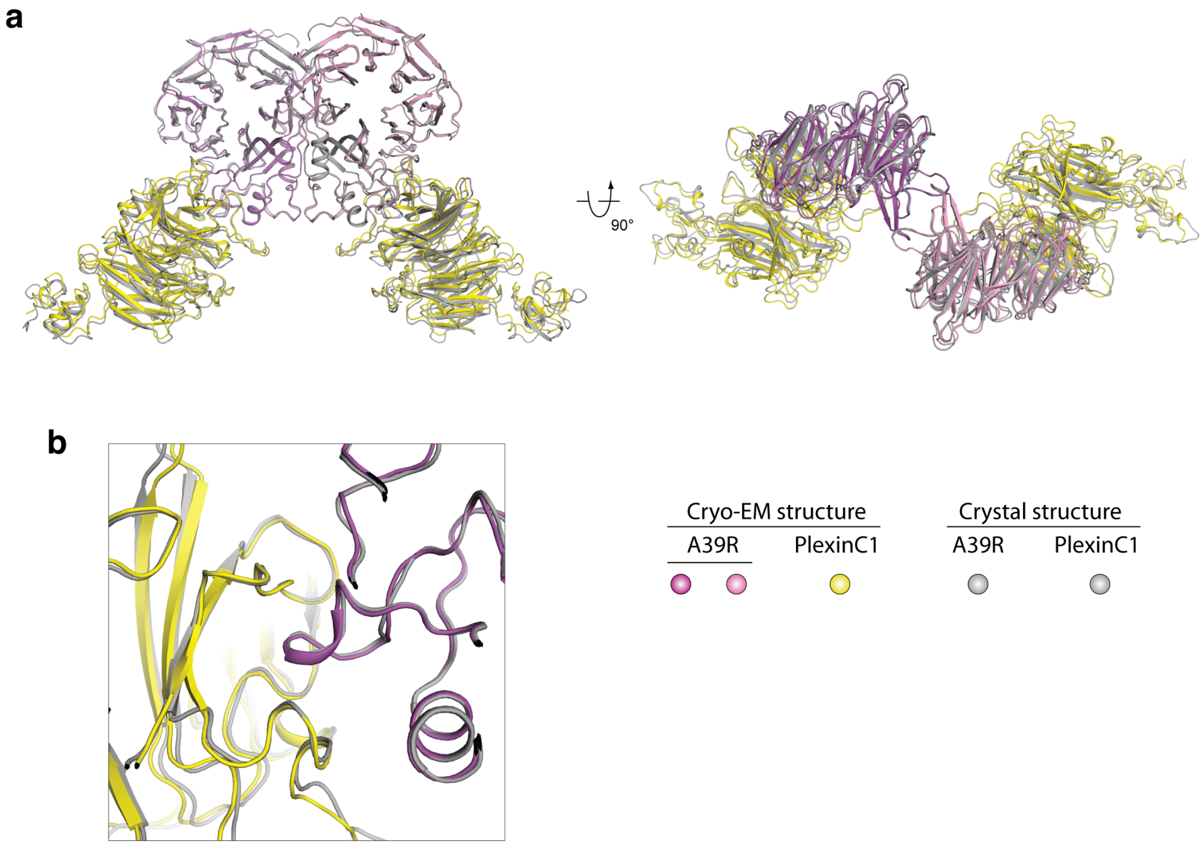

Supplementary Figure 5. Comparison of the cryo-EM structure of the full-length PlexinC1/A39R with the crystal structure of the isolated PlexinC1-Sema-PSI domains in complex with A39R

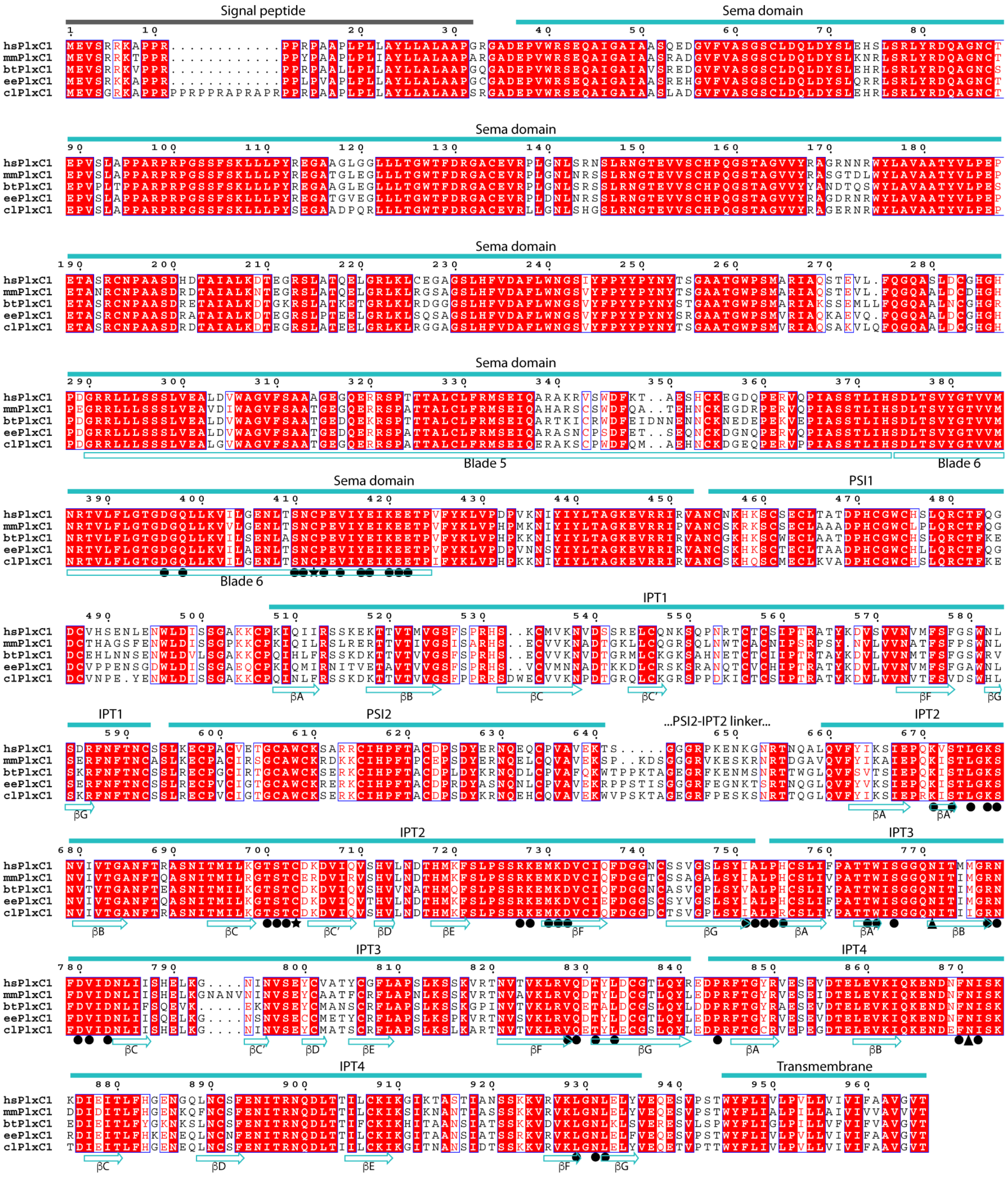

Supplementary Figure 6. Sequence alignment of the extracellular and transmembrane regions of PlexinC1 from several mammalian species. PlexinC1 shows substantial sequence divergence among different mammalian species, particularly in the IPT1 domain and the PSI2-IPT2 linker. However, residues involved in the inter-domain interactions among the Sema, IPT2 and IPT3 domains are conserved. Stars highlight Cys412 and Cys703 that form the inter-domain disulfide bond between the Sema and IPT2 domains. These two cysteine residues are also conserved in lower species such as chicken and zebra fish. Residues involved in non-

covalent interactions among the Sema, IPT2 and IPT3 domains are highlighted with black circles. Black triangles highlight the two glycosylation sites near the IPT2/IPT3 interface. Arrows show the secondary structures of the IPT domains that are identified by the cryo-EM structure. A few secondary structure elements are missing in the final model due to lack of density and therefore not assigned here. Blades 5 and 6 of the Sema domain β-propeller, which are involved in the inter-domain interaction with IPT2-4, are highlighted. Domain boundaries are based on the cryo-EM structure in combination with secondary structure predictions. hs: *Homo sapiens*; mm: *Mus musculus*; bt: *Bos taurus*; ee: *Elephantulus edwardii*; cl: *Canis lupus familiaris*.

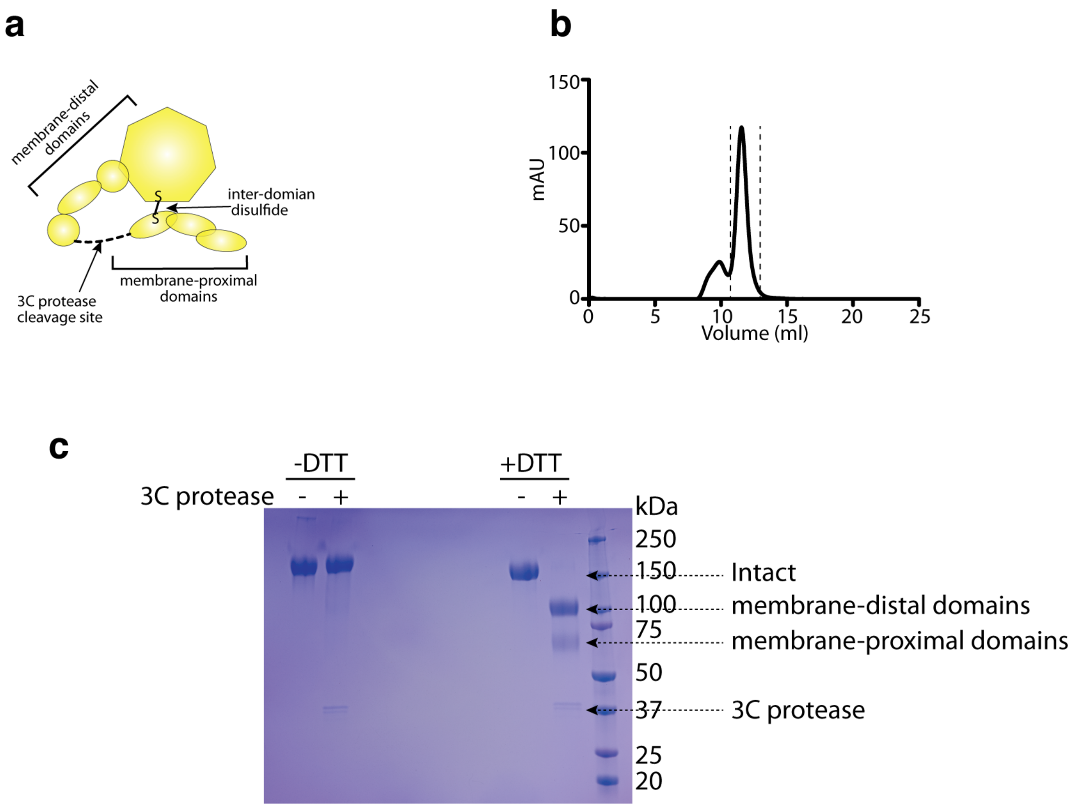

Supplementary Figure 7. Analyses of the inter-domain disulfide bond (C412-C703) in human PlexinC1. (a) Diagram of the human PlexinC1 extracellular region construct. (b) Gel filtration profile of the purified protein. Dashed lines indicate fractions collected for subsequent analyses. (c) SDS gel analyses of the protein. The results show that the purified protein ran as a single band on SDS-PAGE (molecular weight based on the amino acid sequence is ~110 kDa, but appeared to be ~150 kDa because multiple glycosylation sites increase the mass of the protein). Under reducing conditions, 3C protease treatment cleaved the protein into two fragments, corresponding to the N- and C-terminal domains, respectively. In contrast, under non-reducing conditions, the protease-treated protein remained as one band. These results demonstrate that the N- and C-terminal fragments are linked together by an inter-domain disulfide bond. The results in (c) are one experiment from three independent repeats.

Supplementary table 1. Primers used in the study

Primer	Sequence	Comments
hPlxC1KozakM1NheI5	GACTCACTATAGGCTAGCGCCACCATGGAGGTCTCCCGGAGG	5’ primer for a modified pEZT-BM vector
hPlxC1M1568KpnI3	GAACAGAACTTCCAGGCCGCTGCTGGTACCCATCCACTTGCATTTCTTCTTTTC	3’ primer for a modified pEZT-BM vector
A39RXhoI5	GGTTGCGTAGCTGAACTCGAGATCGAATGGCATAAGTTTG	5’ primer for a modified pEZT-BM vector
A39RKpnI3	ATGATGGTGGTGATGGGTACCCATTTTAAGTATTTTTTTCATTCG	3’ primer for a modified pEZT-BM vector
hPlxC1H373AQC5	CATCATCTACCTTGATCGCCTCCGACCTGACATCCG	
hPlxC1H373AQC3	CGGATGTCAGGTCGGAGGCGATCAAGGTAGATGATG	
hPlxC1D396AQC5	ATTCTTGGGGACTGGAGCCGGCCAGTTACTTAAGG	
hPlxC1D396AQC3	CCTTAAGTAACTGGCCGGCTCCAGTCCCCAAGAAT	
hPlxC1C412AQC5	GAATTTGACTTCAAATGCCCCAGAGGTTATCTATG	
hPlxC1C412AQC3	CATAGATAACCTCTGGGGCATTTGAAGTCAAATTC	
hPlxC1E422AQC5	CTATGAAATTAAAGAAGCCACACCTGTTTTCTAC	
hPlxC1E422AQC3	GTAGAAAACAGGTGTGGCTTCTTTAATTTCATAG	
hPlxC1C703AQC5	GAAAGGAACCAGTACCGCCGATAAGGATGTGATAC	
hPlxC1C703AQC3	GTATCACATCCTTATCGGCGGTACTGGTTCCTTTC	
hPlxC1K728EQC5	CTTCCATCAAGCCGGGAGGAAATGAAGGATGTG	
hPlxC1K728EQC3	CACATCCTTCATTTCCTCCCGGCTTGATGGAAG	
PlxC1E860N-K862SQC5	GGTGGACACAGAACTGAACGTGAGCATTCAAAAAGAAAATG	
PlxC1E860N-K862SQC3	CATTTTCTTTTTGAATGCTCACGTTCAGTTCTGTGTCCACC	

hPlxC1M1-5PTY	CTAGAACTAGCCACCGCGGCCGCGCCACCATGGAGGTCTCCCGGAGGAAG	5’ primer for a modified pLVX vector
hPlxC1M1568FLAG3PTY	GCCGCCCTAGATGCAGCTAGCTCACTTGTCGTCATCATCCTTGTAGTCCATCCACTTGCATTTCTTC	3’ primer for a modified pLVX vector
hPlxC1Cla1-F	GCCATCGATATGGAGGTCTCCCGGAGGAAGGCGCCG	
hPlxC1P942 Xba1-R	GCCTCTAGAAGGAACTGACTCCTGCTCGACGTAG	
hPlxC1-3C-F	CTGGAAGTTCTGTTCCAGGGGCCCAACAAGGGGAACAGAACCAACCAGGC	Insertion of HRV 3C cleavage site
hPlxC1-3C-R	CTTGTTGGGCCCCTGGAACAGAACTTCCAGCTCCTTGGGTCTTCCTCCTCCTGATG	Insertion of HRV 3C cleavage site
hPlxC1 939insert-F	GAGCTCTACGTCGAGCAGGAGGGCTCCTCAGGCTCAGTTCCTTCCACATGGTATTTTC	
hPlxC1 939insert-R	GAAAATACCATGTGGAAGGAACTGAGCCTGAGGAGCCCTCCTGCTCGACGTAGAGCTC	
hPlxC1 938del-F	GGAAACCTGGAGCTCTACGTCGAGCCTTCCACATGGTATTTTCTGATTG	
hPlxC1 938del-R	CAATCAGAAAATACCATGTGGAAGGCTCGACGTAGAGCTCCAGGTTTCC	
